# The Impact of Age on the Lipidomic Profile of the Stratum Corneum and Associated Effects on Structure, Function and Overall Skin Health in Adults Predisposed to Atopic Dermatitis

**DOI:** 10.1111/exd.70192

**Published:** 2025-12-17

**Authors:** S. F. Williams, P. Andrew, K. Brown, J. Chittock, A. Pinnock, A. Poyner, M. J. Cork, S. G. Danby

**Affiliations:** ^1^ Sheffield Dermatology Research, Division of Clinical Medicine, School of Medicine & Population Health University of Sheffield Sheffield UK; ^2^ The Paediatric Dermatology Clinic Sheffield Children's Hospital Sheffield UK

## Abstract

Advancing age is associated with an increasing prevalence of dry skin conditions such as xerosis, asteatotic eczema and atopic dermatitis (AD). Although broad changes in stratum corneum (SC) lipids and AD history have been implicated, age‐related alterations in the SC lipidome within at‐risk populations remain unclear. We characterised SC structure and lipidomic profiles in 58 adults with dry, eczema‐prone skin across a wide age range. Assessments included visual dryness, biophysical properties (TEWL, capacitance, skin‐surface‐pH), irritant sensitivity, ATR‐FTIR spectroscopy and lipidomic analysis through quantification of extracted SC lipids via mass spectrometry. Age correlated significantly with increased dryness (*r* = 0.46, *p* ≤ 0.0001) and reduced hydration (r = −0.42, *p* ≤ 0.0001). Spectroscopy revealed declines in total lipids (*p* < 0.0026), water (*p* < 0.0009), lipid esters (*p* < 0.0001) and carboxylates (*p* < 0.0004) with age. Among 1385 quantified lipid species, triacylglycerol (TAG) was most abundant; TAG 46:1;0 associated with dryness (r = −0.42, *p* ≤ 0.0001). Ceramides CER[AH] (*p* < 0.0001), CER[AP] (*p* < 0.0001), CER[AdS] (*p* = 0.042), CER[NP] (*p* = 0.031) and CER[NdS] (*p* < 0.0001) all significantly increased with age relative to protein. Notably, CER[NdS] species shifted towards shorter (16ºC) acyl chains (+2.23%, *p* = 0.01) and away from longer (24ºC) chains (−3.9%, *p* < 0.0001). The CER[NdS]/CER[NH] ratio correlated with age (*r* = 0.59, *p* < 0.0001), dryness (*r* = 0.36, *p* = 0.0006), and barrier integrity (*r* = 0.59, *p* < 0.0001) (all *p* ≤ 0.0006). Within an at‐risk population, SC lipid levels change as the skin ages. These changes, especially an increase in short acyl chain NdS ceramides, were associated with the decline in skin barrier function and may help explain the increased prevalence of xerosis and the (re)emergence of eczema in later life.

## Graphical abstract

Advancing age in eczema‐prone adults correlates with increased dryness, reduced hydration and altered lipid profiles. Spectroscopy showed declines in lipids, water, esters and carboxylates. Lipidomics revealed rising ceramide species, shorter acyl chains and TAG associations. These changes impair barrier integrity, aiding in the explanation of xerosis and eczema prevalence in later life.

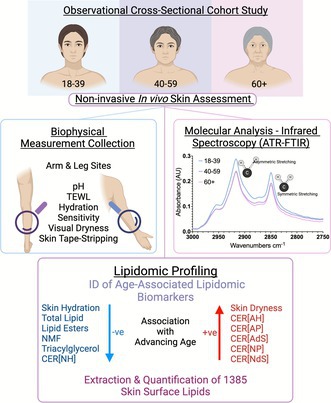

## 1 Introduction

The stratum corneum (SC) forms the outermost layer of the skin, providing a physical barrier between the body and the external environment, whilst preventing excessive moisture loss. Skin xerosis can occur because of defects within the SC structure and is strongly associated with increasing age and previous history of atopic dermatitis (AD), where the innate healthy SC structure has been impaired [1]. The prevalence of xerosis in the elderly is high, affecting between 30% and 75% depending on the population and setting [1]. Older age is also associated with a higher prevalence of AD compared to younger adults (11.6 vs. 7.7%) [2]. This is due to the re‐emergence of previously resolved childhood AD and the development of new cases (late‐onset AD). The age‐associated changes to the skin that increase the risk of AD and xerosis more generally are poorly understood [3].

SC lipid depletion is an important structural change that has been documented to occur with advanced age and is associated with increasing skin surface pH and colonisation by potentially pathogenic bacteria such as *Staphylococcus aureus*, which further contributes to skin barrier degradation [4, 5]. In particular, broadly altered ceramide levels within the lipid lamellae appear to be linked to dry‐skin conditions (like AD), which increase SC permeability, causing greater transepidermal water loss (TEWL) and xerosis incidence [6, 7]. There are a plethora of ceramide subspecies within the SC, some with purported positive effects on the formation of the extracellular lipid matrices and cellular function and others with negative effects, so it is important to characterise these changes in more detail.

This study aimed to noninvasively characterise the SC structure and lipidomic profile of adults over a broad age range with dry, eczema‐prone skin. The lipidomic profiling proposed in this study was far more comprehensive than observed in currently available literature, with the hope of identifying age‐associated biomarkers of xerosis and skin barrier dysfunction. Identification of structural alterations associated with age could aid the development of treatments, highlighting ingredients to be included in topical formulations to combat and reduce the incidence of dry‐skin conditions in ageing populations.

## 2 Patients and Methods

### 2.1 Study Design and Setting

The results presented in this work consist of the baseline measurements (before the treatment period began) taken as part of the BRaCE study (An investigation of the skin barrier reinforcing effects of a ceramide cream). It was a double‐blind participant‐controlled interventional study of 58 adult participants with dry, eczema‐prone skin, defined by previous self‐determined incidence of eczema (formal medical history was not requested, but skin TEWL and dryness thresholds were applied at participant screenings to confirm skin barrier defect consistent with previous eczema incidence, as detailed in the Supporting Information). The results of the trial are presented elsewhere [8]. Herein, only the baseline data is presented following stratification into three age groups: 18–39, 40–59 and 60+ years, so that the impact of ageing on skin structure and lipid composition can be evaluated.

The study was given ethical approval by the University of Sheffield Research Ethics Committee (UREC)—study reference number: 044518. All subjects were included on a voluntary, first‐come, first‐served basis and provided informed consent prior to participation. The study was performed in accordance with the Helsinki Declaration of 1964 and later amendments. Full inclusion and exclusion criteria and further study information are provided in the Supporting Information.

### 2.2 Skin Assessments

Skin biophysical measurements and visual assessments were collected from volar forearm and lateral lower leg sites from each participant in a temperature and humidity‐controlled room (20°C ± 2°C and 45% ± 10% relative humidity) at the Skin Barrier Facility, Sheffield Dermatology Research. Skin assessments and measurements taken were: visual skin dryness [9], skin hydration (Corneometer), TEWL (Aquaflux), skin surface pH, sensitivity testing (erythema pre‐post SLS occlusion), skin tape‐stripping and subsequent quantification of extracted skin lipids via mass spectrometry, skin surface molecular structure via attenuated total reflectance infrared spectroscopy (ATR‐FTIR). Full details on skin visual assessments and biophysical measurements are provided in the Supporting Information.

### 2.3 Outcomes

The outcomes of this part of the observational cross‐sectional cohort study included the differences in the following properties between the three age groups: (i) visual dryness and biophysical properties (transepidermal water loss [TEWL], capacitance and skin surface pH); (ii) molecular composition assessed through ATR‐FTIR spectroscopy; and (iii) the profiles of medium‐long chain lipid species present within the SC, extracted via skin tape strip (STS) and quantified through orbitrap mass spectrometry (lipidomic analysis). Full details of skin assessments and sample analysis can be found in the Supporting Information.

#### 2.3.1 Nomenclature

Ceramides (CER) are referred to using established nomenclature based upon abbreviations for their acyl chain (N‐, A‐, Eo‐) followed by the sphingoid base (SB) [10]. For the identification of individual species, the class abbreviation is followed by the number of carbons, the number of double bonds, and the number of hydroxyl groups, for the SB, followed by the acyl chain. AdS17:0;2/19:0;1 denotes an AdS ceramide with 17 carbons, zero double bonds and two hydroxyl groups in the SB; 19 carbons, zero double bonds and one hydroxyl group in the acyl chain.

#### 2.3.2 Statistical Analysis

All data were analysed using Prism nine (GraphPad Software, La Jolla, USA). Comparisons between age groups were made using a one‐way or two‐way analysis of variance and a two‐stage step‐up Benjamini, Krieger and Yekutieli multiple comparison correction method. One‐way or two‐way analysis was chosen depending on whether the data were stratified via age or via age and lipid species. The significance threshold was *p* < 0.05. Results are presented as mean ± SD unless otherwise stated. Linear models were computed using the built‐in linear regression and linear discriminant analysis functions in R (RStudio, Version 2024.04.0 + 735, Posit Software, USA).

## 3 Results

Out of the 95 adults who volunteered, a total of 58 participants consented and were enrolled in the study between April 2022 and March 2023. Upon inclusion, participants were stratified into three study groups based on their age: 18–39, 40–59 and 60+. Background information on the study cohort is displayed in Table 1.

**TABLE 1:** Cohort demographics.

Demographic	Set analysis			
Age group	18–39	40–59	60+	All participants
*n*	24	16	18	58
Sex				
Male	8	4	12	24
Female	16	12	6	34
Ethnicity				
Asian—Indian	1			1
Asian—Pakistani		1		1
Black—Caribbean			1	1
Chinese	2	1		3
Mixed—White/Asian		1		1
White—British	20	12	16	48
White—Irish			1	1
White—Other	1	1		2
Age				
Mean ± SD (Min, Max)	26.71 ± 6.91 (18, 39)	46.87 ± 5.22 (40, 57)	72.50 ± 7.86 (61, 88)	46.47 ± 20.75 (18, 88)
Fitzpatrick skin type				
I	1	2	1	4
II	16	6	8	30
III	4	5	8	17
IV	3	3	0	6
V	0	0	1	1
VI	0	0	0	0
Median (Min, Max)	2.00 (1, 4)	2.00 (1, 4)	2.50 (1, 5)	2.00 (1, 5)
Age of eczema onset				
Under 2	12	5	5	22
2–5	7	2	2	11
6–10	1	0	1	2
11–17	2	2	0	4
18+	2	7	10	19
Last symptom (itchy skin) occurrence				
Itchy skin ever:	24	16	18	58
In last year:	22	13	16	51
In last week:	17	10	10	37

As the primary focus of this work is on the impact of age, quoted mean values include both male and female participants from each age group, with two‐way ANOVAs used to assess the impact of age, sex and the age‐sex interaction on results.

**FIGURE 1:**
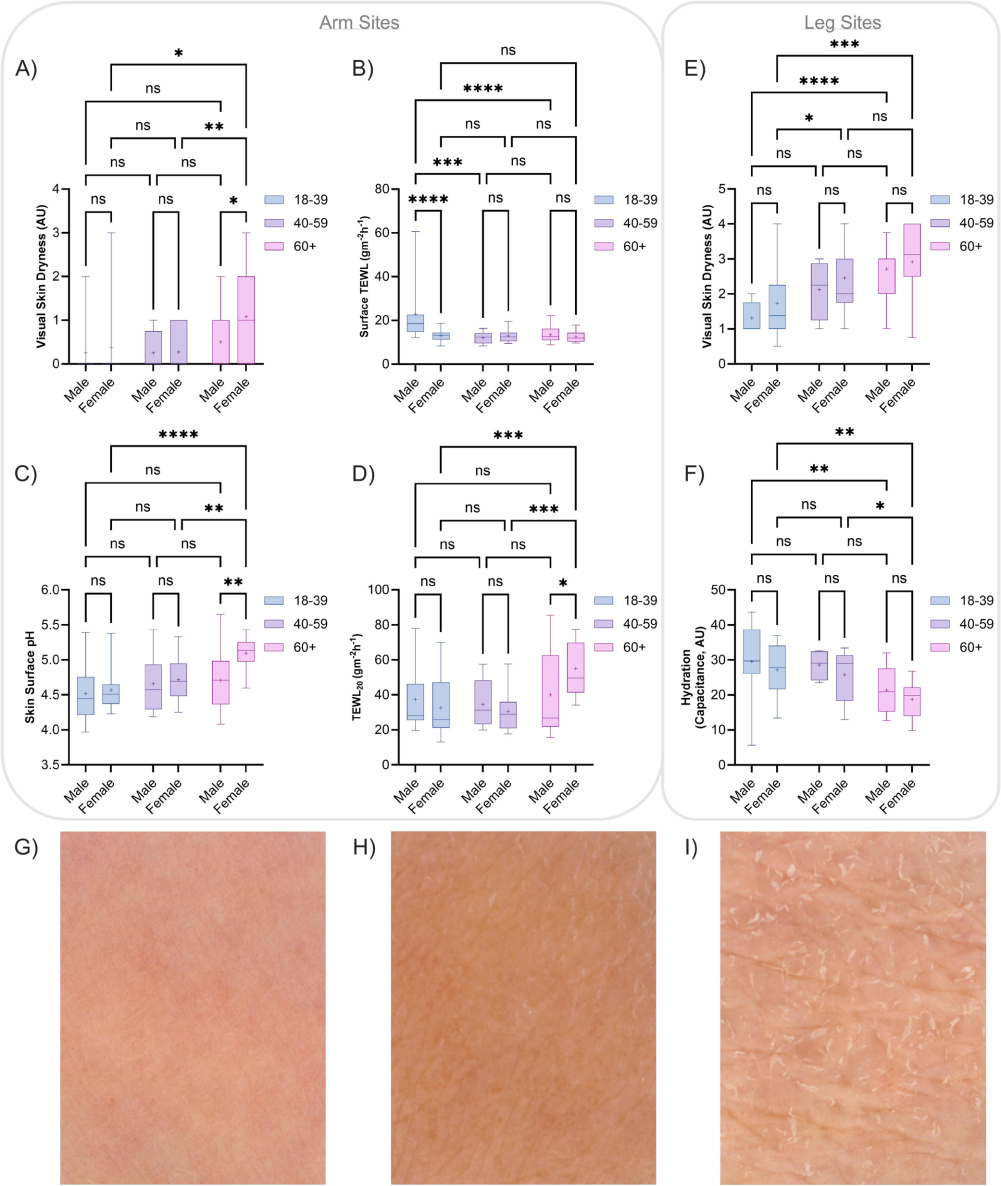
Baseline skin assessment of volar forearm (A–D) and leg (E, F) sites, stratified by age group and sex. Overall dry skin (ODS) score was used as a visual measure of dryness (A, E), with skin function measured as transepidermal water loss on the skin surface (TEWL—B) and after removal of 20 tape‐strips (TEWL_20_—D), skin surface pH (C) and capacitance (F) collected as biophysical measurements. Example clinical images of leg skin sites from participants aged 18–39 (G), 40–59 (H) and 60+ (I) who were given an ODS score of 1.5, 2 and 4 respectively. Box‐and‐whisker plots depict the minimum, lower quartile, median, upper quartile and maximum values present in each group, with ‘+’ indicating the mean value. Asterisks indicate the results of one‐way ANOVA with post hoc Tukey HSD statistical testing (ns = not significant, *p < 0.05, **p < 0.01, ***p < 0.001, ****p < 0.0001).

Visual assessments of skin surface dryness (xerosis) increased with age on both arm (18–39: 0.33 ± 0.80 vs. 60+: 0.67 ± 0.82 AU) and leg sites (18–39: 1.59 ± 0.82 vs. 60+: 2.78 ± 0.91 AU), and age was the only significant factor in dryness variance on both forearm (*p* = 0.011) and leg sites (*p* < 0.0001). Surface biophysical measurements collected showed increased skin‐surface‐pH (18–39: 4.55 ± 0.32 vs. 60+: 4.84 ± 0.40) and decreased skin hydration (18–39: 28.00 ± 8.65 vs. 60+: 20.52 ± 6.27 AU) with advancing age, with both age (*p* < 0.0001) and sex (*p* = 0.019) significant in pH change whilst only age (*p* < 0.0001) was a significant factor in hydration variance. Skin barrier function (TEWL) was not significantly different between the youngest and the eldest groups on average. However, due to a greater TEWL in the youngest males, age (*p* = 00017), sex (*p* = 0.0079) and age‐sex interaction (*p* = 0.00042) were significant factors. TEWL after 20 STS (TEWL_20_) was highest in the 60+ group (18–39: 34.17 ± 17.84 vs. 60+: 45.02 ± 21.51 g m^−2^ h^−1^), indicative of reduced skin barrier integrity. Age (*p* = 0.0020) was significant in TEWL_20_ variance (Figure 1). Although participants aged 60+ exhibited a greater baseline skin redness (erythema index), skin sensitivity to SLS (1%, applied under an occlusive patch) was similar in all age groups (Figure S1).

**FIGURE 2:**
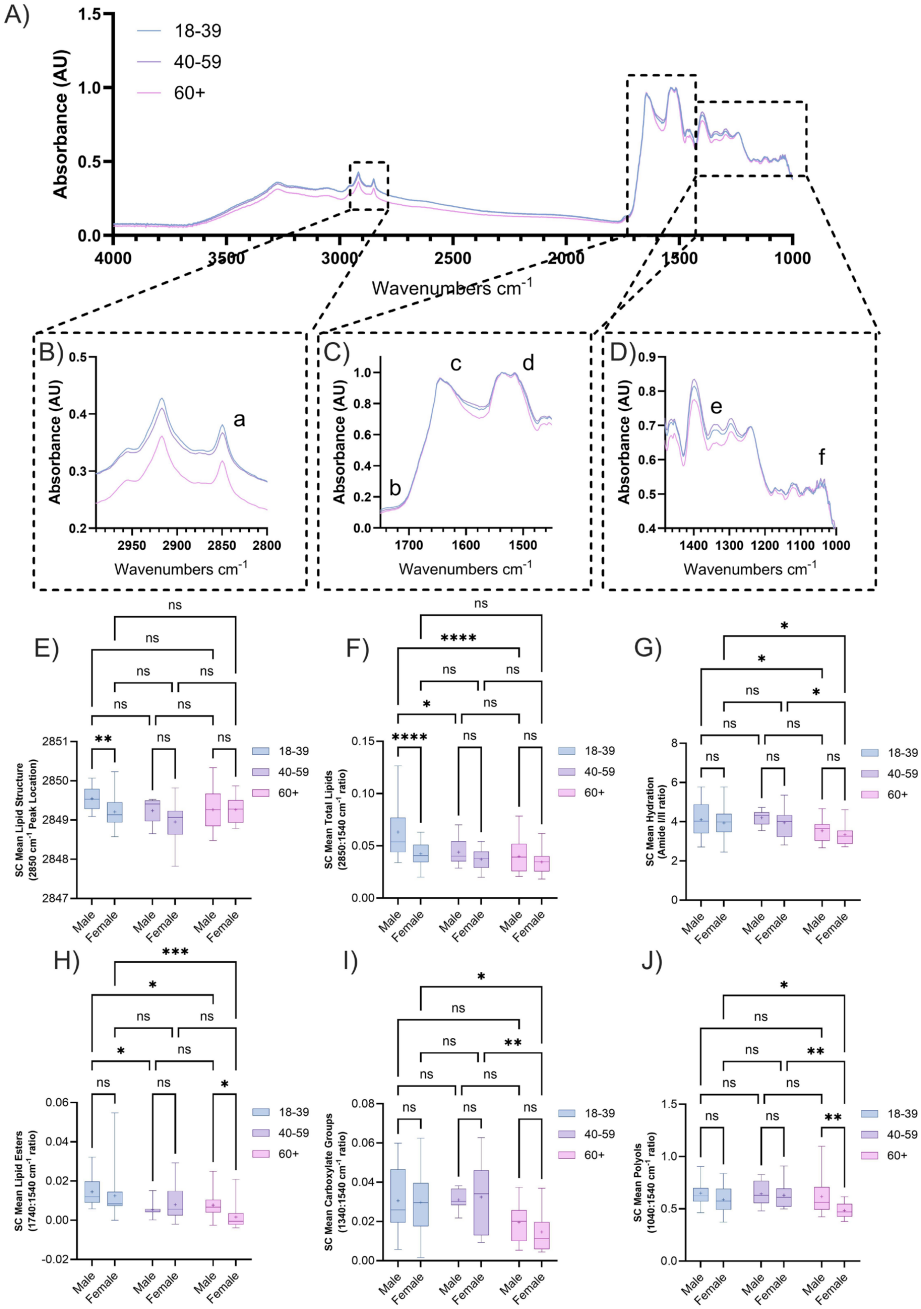
Average mid‐infrared spectrum of in vivo human skin collected just beneath the surface (after removal of five skin tape‐strips) of volar forearm skin sites stratified via age (A–D), normalised to Amide II region area. Callouts highlight observed spectral differences in regions containing peaks associated with lipids (B: 2800–2990 cm‐1), amide I (and water) and II (C: 1480–1700 cm‐1) and carboxylates within the fingerprint region (D: 1000–1440 cm‐1). Highlighted peaks correspond to functional groups indicative of (a) total lipid, (b) lipid esters, (c) amide I (contributed to by water), (d) amide II, (e) carboxylates and (f) polyols. Stratum corneum mean results depict peaks associated with lipid structure (E), total lipid (F), hydration (G), lipid esters (H), carboxylates (I), polyols (J) from a depth of 0–6 μm, stratified by age and sex. Box‐and‐whisker plots depict the minimum, lower quartile, median, upper quartile and maximum values present in each group, with ‘+’ indicating the mean value. Asterisks indicate the results of one‐way ANOVA with post hoc Tukey HSD statistical testing (ns = not significant, *p < 0.05, **p < 0.01, ***p < 0.001, ****p < 0.0001).

ATR‐FTIR spectroscopy was utilised to identify alterations in chemical functional groups between the cohorts. Visual inspection of average spectra (Figure 2A) collected from just beneath the SC surface (STS 5) highlighted differences in SC lipid (methylene, ‐CH_2_), water (hydroxyl, ‐OH) and natural moisturising factors (NMF) measured as carboxylate (RCOO) levels with age (Figure 2B–D). Due to the shallow penetration depth of ATR‐FTIR spectroscopy into skin (1–2 μm), spectra collected after 5, 10, 15 and 20 STS were averaged together (SC Mean) to depict SC structural features up to 6 μm from the surface. Total lipids (18–39: 0.049 ± 0.020 vs. 60+: 0.038 ± 0.015), hydration (18–39: 3.99 ± 0.81 vs. 60+: 3.47 ± 0.53), lipid esters (18–39: 0.013 ± 0.010 vs. 60+: 0.006 ± 0.007), and carboxylates (18–39: 0.030 ± 0.017 vs. 60+: 0.018 ± 0.010) all decreased with age. Age was significant in the variance of hydration (*p* = 0.0006), lipid esters (*p* < 0.0001) and carboxylates (*p* = 0.0003), whereas both age (*p* < 0.0001) and sex (*p* = 0.0007) were significant factors in total lipid change (Figure 2F–I). Although polyol levels did not drop on average between the youngest to eldest groups (18–39: 0.61 ± 0.12 vs. 60+: 0.57 ± 0.16), the observed decrease in polyols in 60+ Females resulted in both age (*p* = 0.024) and sex (*p* = 0.0092) being significant factors in polyol variance (Figure 2J). Lipid chain conformation, indicated by the position of the lipid peak at 2850 cm^‐1^ [11, 12], was more orthorhombic (tightly packed) in the 40–59 age group compared to either of the other two groups (indicated by a lower wavenumber position), and both age (*p* = 0.029) and sex (*p* = 0.015) were found to be significant contributors to lipid structure (Figure 2E). Full structural information from surface to STS20 is provided in the Supporting Information (Figures S2 and S3).

**FIGURE 3:**
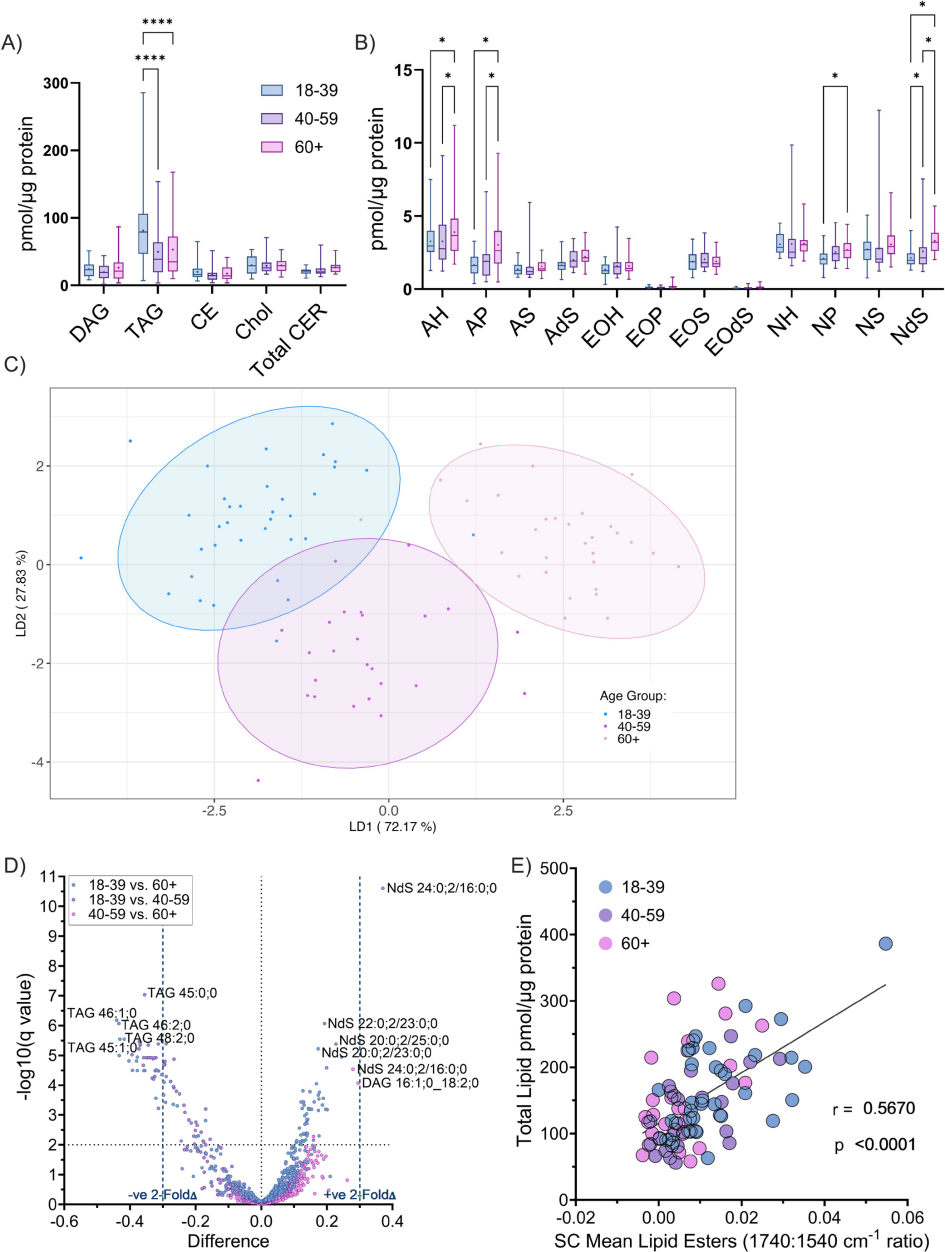
Top‐level lipidomic analysis extracted from second skin tape‐strip (STS) removed from volar forearm sites; Age‐associated changes in stratum corneum lipid groups (A) and ceramide (B) levels, relative to protein. Individual species analysis through linear discriminant analysis with age group ellipses set at 95% confidence interval (C) and individual t‐test comparison of all lipid subspecies within each age group, depicted as a volcano plot, with blue dotted lines indicating the threshold for a twofold difference in age group comparison. Difference values equate to lipid amount in the older group minus lipid amount in the younger group, with the legend indicating age groups compared (D). Pearson association between total lipid quantified through STS extraction and ATR‐FTIR derived lipid ester quantification shown as a scatter plot (E). Box‐and‐whisker plots depict the minimum, lower quartile, median, upper quartile and maximum values present in each group, with ‘+’ indicating the mean value. Asterisks indicate the results of two‐way ANOVA with post hoc Tukey HSD statistical testing (ns = not significant, *p < 0.05, **p < 0.01, ***p < 0.001, ****p < 0.0001).

Lipidomic analysis was conducted through quantification of extracted lipids collected from the SC during tape‐stripping (STS discs 2 and 5). A total of 1385 lipids were detected in the samples from STS 2 and 1234 from STS 5. Findings were similar for both samples, so only the data from STS 2, with the greater diversity of lipids, is presented for brevity. Figure 3A shows the breakdown of lipid species detected, which is also stratified by sex in the Supporting Information (Figure S4). Triacylglycerol (TAG) was the most abundant lipid species, and there was a significant depletion (18–39: 81.43 ± 52.31; vs. 60+: 53.16 ± 43.87 *p* < 0.0001; vs. 40–59: 49.50 ± 39.52 pmol/μg protein, *p* < 0.0001) of TAGs in those above 39, compared to younger individuals (Figure 3A). When breaking down ceramide sub‐classes: CER[AH] (18–39: 3.27 ± 1.30 vs. 60+: 3.90 ± 1.87 pmol/μg protein, *p* < 0.0001), CER[AP] (18–39: 1.66 ± 0.75 vs. 60+: 3.03 ± 1.79 pmol/μg protein, *p* < 0.0001), CER[AdS] (18–39: 1.64 ± 0.50 vs. 60+: 2.21 ± 0.64 pmol/μg protein, *p* = 0.042), CER[NP] (18–39: 2.09 ± 0.57 vs. 60+: 2.70 ± 0.76 pmol/μg protein, *p* = 0.031) and CER[NdS] (18–39: 2.14 ± 0.58 vs. 60+: 3.30 ± 0.89 pmol/μg protein, *p* < 0.0001) all significantly increased with age relative to protein (Figure 3B). There was only a weak correlation between total extracted lipids and ATR‐FTIR‐derived total lipid levels relative to protein (*r* = 0.22—Figure S5), but a stronger association between total extracted lipids and ATR‐FTIR‐derived lipid esters (*r* = 0.57 Figure 3E). This is explained by the fact that our lipidomic analysis focuses on medium‐long chain lipid species, dominated by lipid esters, and excludes short‐chain lipids (i.e., free fatty acids).

Further analysis was undertaken to identify changes in specific lipid subspecies, such as carbon chain length. Not all subspecies were present in all samples; therefore, they were only included in the analysis when detected in at least 75% of participants within at least one group; any remaining missing values were imputed with the median value of the group. After excluding subspecies falling below the 75% threshold, 382 subspecies remained. Linear discriminant analysis (LDA) showed a significant link between quantified lipid subspecies and age via complete separation of the 60+, 95% CI ellipse from younger age groups (Figure 3B). Plot loadings indicating the association between lipid subspecies and the linear discriminants used to separate age groups are supplied in a Supporting Information. Individual t‐tests, presented as a volcano plot (Figure 3D), were used to identify subspecies significantly changing between all age groups. NdS 24:0;2/16:0;0 showed the greatest increase between 18–39 and 60+ (Mean Diff.: 0.37 ± 0.039 log_10_ pmol/mg protein, *p* < 0.0001), whereas TAG 46:1;0 decreased significantly with age (Mean Difference: −0.44 ± 0.064 log_10_ pmol/mg protein, *p* < 0.0001). Further details of the t‐test results are tabulated in the Supporting Information (Tables S1 and S2). A linear regression model was used to predict participant age, with a high correlation (*r* = 0.62, *p* < 0.0001) observed between predicted and actual ages (mean prediction error: 11.77 ± 1.98 Years—Figure S6).

**FIGURE 4:**
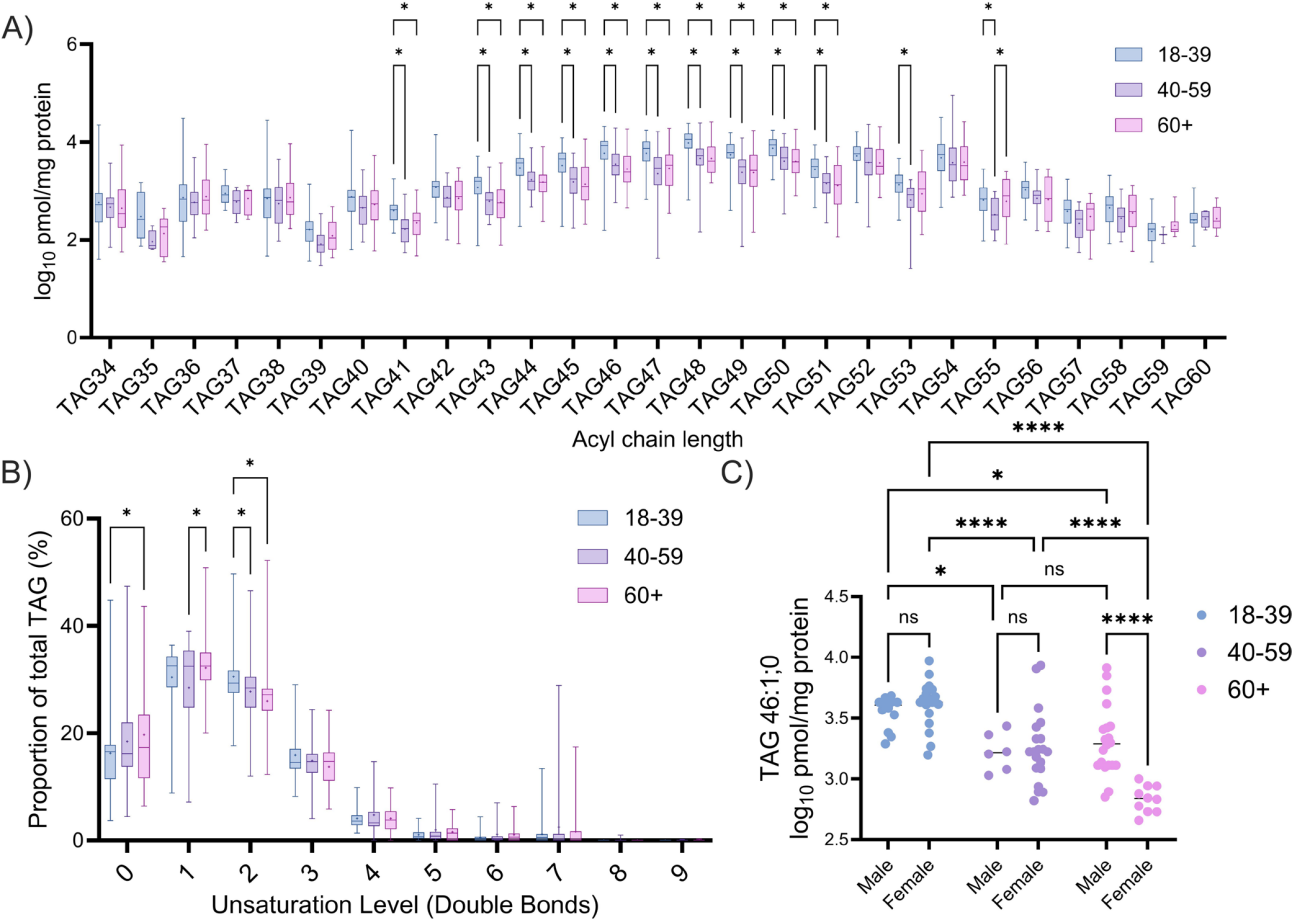
Triacylglycerol (TAG) association with age via comparison of aggregated TAG species of sphingosine carbon chain length from 34ºC–60ºC (A), proportion of unsaturation level (B), and TAG 46:1;0 (C), also stratified by sex. Box‐and‐whisker plots depict the minimum, lower quartile, median, upper quartile and maximum values present in each group, with ‘+’ indicating the mean value. Asterisks indicate the results of two‐way (A and B) or one‐way (C) ANOVA with post hoc Tukey HSD statistical testing (ns = not significant, *p < 0.05, **p < 0.01, ***p < 0.001, ****p < 0.0001).

As TAG and CER[NdS] subspecies had shown strong links to age, changes within these ceramide classes were characterised further. Many TAG groups were observed to deplete with age when stratified by carbon chain length (Figure 4A). The proportion of fully saturated TAGs significantly increased with age (18–39: 16.23 ± 8.09 vs. 60+: 19.7110.33%, *p* = 0.004—Figure 4B). TAG 46:1;0 is one of the most abundant TAGs and decreased (18–39: 3.59 ± 0.16 vs. 40–59: 3.25 ± 0.27 vs. 60+: 3.15 ± 0.33 log_10_ pmol/mg protein) in individuals older than 39, with age (*p* < 0.0001), sex (*p* = 0.023) and age‐sex interaction (*p* < 0.0001) all significant in variance (Figure 4C).

**FIGURE 5:**
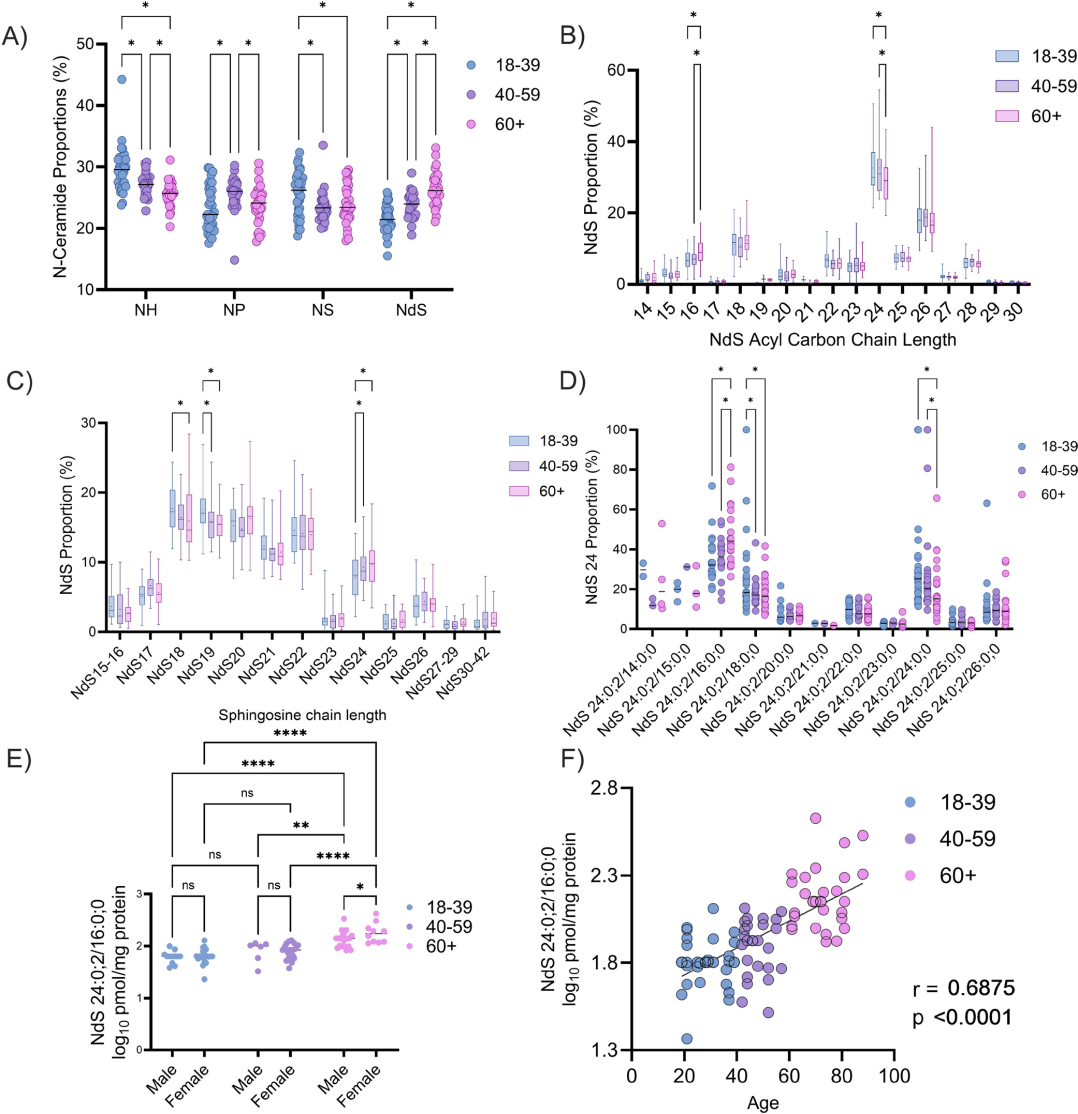
Overall N‐ceramide and CER[NdS] association with age via comparison of: N‐ceramide proportions (A), CER[NdS] acyl chain length from 14ºC–30ºC as a proportion of total CER[NdS] (B), proportion CER[NdS] species of sphingosine carbon chain length from 15ºC–42ºC (C), individual CER[NdS] species of 24ºC sphingosine chain length from acyl chain length 14ºC–26ºC (D), NdS 24:0;2/16:0;0 plotted by age group (E—also stratified by sex) and Pearson association with age as a continuous variable (F). Box‐and‐whisker plots depict the minimum, lower quartile, median, upper quartile and maximum values present in each group, with ‘+’ indicating the mean value. Asterisks indicate the results of two‐way (A–D) or one‐way (E) ANOVA with post hoc Tukey HSD statistical testing (ns = not significant, *p < 0.05, **p < 0.01, ***p < 0.001, ****p < 0.0001).

As a proportion of total N‐ceramides, CER[NdS] increased (18–39: 21.52 ± 2.29 vs. 60+: 26.44% ± 2.78%, *p* < 0.0001) in the skin with age (Figure 5A), mirroring the change in CER[NH]. Within the NdS class, the proportion of NdS lipids with longer acyl chain lengths decreased with age, with the abundance of 24‐carbon acyl chains reducing (18–39: 32.63 ± 7.72 vs. 60+: 28.73% ± 5.40%, *p* < 0.0001) compared to an increase (18–39: 6.76 ± 3.02 vs. 60+: 8.99% ± 4.00%, *p* = 0.01) in 16‐carbon chains (Figure 5B). The abundance of 24‐carbon SB chain length increased significantly (18–39: 7.87 ± 3.19 vs. 60+: 9.70% ± 3.51%, *p* = 0.0054, Figure 5C), whereas 18‐ and 19‐carbon chains decreased. When looking specifically at NdS subspecies with a 24ºC SB, the 16‐carbon acyl chain increased significantly (18–39: 34.71 ± 11.43 vs. 60+: 45.52% ± 13.64%, *p* = 0.0023, Figure 5D), and was largely compensated by a drop in 24‐carbon acyl chain abundance. In absolute terms, NdS 24:0;2/16:0;0 increased (18–39: 1.80 ± 0.14 vs. 60+: 2.17 ± 0.17 log_10_ pmol/mg protein) with age and only age was found to be a significant factor of variance (*p* < 0.0001—Figure 5E), validated by a strong linear correlation with age (*r* = 0.69—Figure 5F).

**FIGURE 6:**
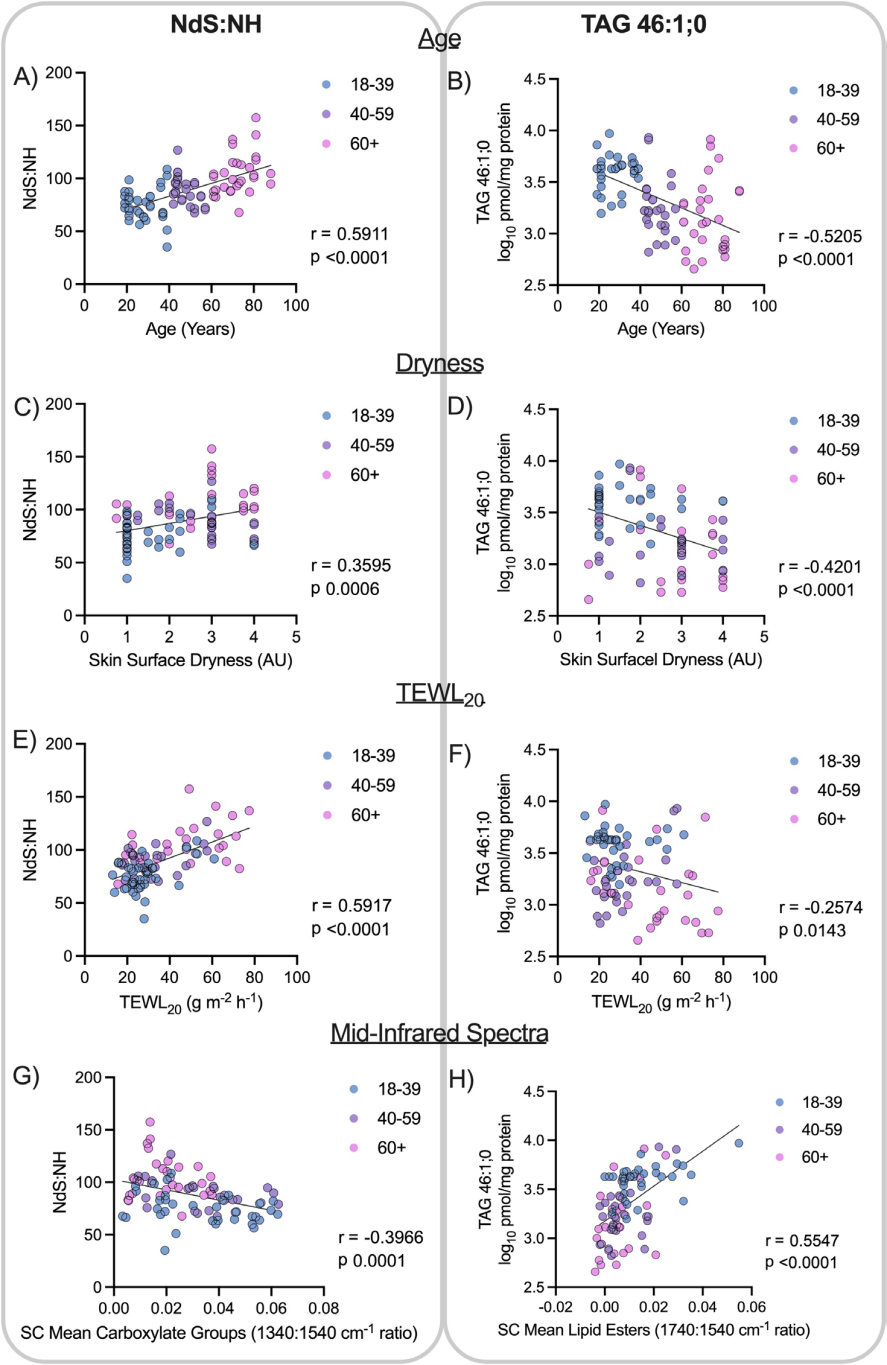
Pearson Correlations represent functional associations between CER[NdS]: CER[NH] or TAG 46:1;0 with age (A, B), skin surface dryness (C, D), and skin function—TEWL_20_ (E, F). Mid‐infrared regions associated with carboxylates and lipid esters were correlated with CER[NdS]: CER[NH] (G) and TAG 46:1;0 respectively (H).

Given the opposing association of CER[NH] and CER[NdS] with age (Figure 5A), the ratio between the two ceramide classes was determined to combine the effects into a single metric and associated with age (*r* = 0.59), TEWL_20_ as a marker of skin barrier dysfunction (*r* = 0.59) and skin dryness (r = 0.36). TAG 46:1;0 was also associated with age (r = −0.52) and skin dryness (r = −0.42) but not TEWL_20_ (r = −0.26, Figure 6). Further exploration of the associations between changes in skin composition and function is presented in Table 2. The strongest factors associated with TEWL_20_ were the levels of polyols (like glycerol), the NH/NdS ceramide ratio, and the levels of carboxylate groups. In contrast, the strongest factor associated with skin surface dryness was the levels of lipids (total).

**TABLE 2:** Association between Age, TEWL_20_ and skin dryness with the molecular composition and structure of the SC.

Feature	Age		TEWL_20_		Dryness	
	Pearson r	*p*	Pearson r	*p*	Spearman r	*p*
Age			0.38	*p* < 0.001	0.46	*p* < 0.0001
TEWL_20_	0.38	*p* < 0.001			0.36	*p* < 0.001
Dryness	0.46	*p* < 0.0001	0.41	*p* < 0.0001		
Hydration ^a^	−0.42	*p* < 0.0001	−0.18	0.09	−0.53	*p* < 0.0001
Skin‐surface‐pH	0.31	*p* < 0.01	0.24	*p* < 0.05	0.29	*p* < 0.05
Skin sensitivity ^b^	0.41	*p* < 0.0001	0.35	*p* < 0.001	0.23	*p* < 0.05
Lipid structure ^c^	−0.05	0.61	0.37	*p* < 0.001	−0.07	0.53
Total lipid ^c^	−0.24	*p* < 0.05	−0.01	0.95	−0.55	*p* < 0.0001
Lipid esters ^c^	−0.40	*p* < 0.001	−0.13	0.23	−0.35	*p* < 0.001
Polyols ^c^	−0.22	*p* < 0.05	−0.66	*p* < 0.0001	−0.38	*p* < 0.001
Hydration (AI/II) ^c^	−0.31	*p* < 0.01	−0.43	*p* < 0.0001	−0.43	*p* < 0.0001
Carboxyl groups ^c^	−0.35	*p* < 0.001	−0.55	*p* < 0.0001	−0.43	*p* < 0.0001
NdS:NH ratio ^d^	0.59	*p* < 0.0001	0.59	*p* < 0.0001	0.34	*p* < 0.01
TAG 46:1;0 ^d^	−0.52	*p* < 0.0001	−0.26	0.21	−0.42	*p* < 0.0001

*Note:* ^a^Measured as capacitance, ^b^Change in erythema index in response to SLS patch testing, ^c^Derived from ATR‐FTIR spectra (lipid structure, total lipid, lipid esters, polyol, hydration, carboxyl groups). ^d^Derived from the lipidomic analysis of SC samples.

## 4 Discussion

The natural ageing process affects tissues' and organs' reparative capabilities throughout the body, but given the skin's function as the external barrier to the environment, the organ is subject to extrinsic factors such as ultraviolet (UV) radiation, cigarette smoke and pollution, which exacerbate the intrinsic factors of ageing (time, hormone regulation, genetics) [13]. It is well documented that the prevalence of AD is greatest in infants, usually reported at around 20% incidence, with studies observing up to 30% in those aged 0–12 months [14, 15]. AD incidence decreases with age, but as the skin's regenerative capabilities diminish, it becomes more susceptible to damage [16], and the prevalence of dry skin conditions such as AD then increases. One study reported a 6% increase per year in the odds ratio of developing AD as adults aged, based on the health records of 9.1 million participants [2]. Mid‐infrared spectroscopic and lipidomic profile analysis of skin allows noninvasive quantification of biomarkers linked to skin function and identification of the SC structural changes that contribute to the functional alterations of aged skin.

### 4.1 Advancing Age Was Associated With Increased Skin Dryness and pH, Whilst Barrier Function Diminished

An increase in skin dryness was observed with age; assessed visually, based on capacitance (leg sites) and confirmed by direct quantification of SC water content (by ATR‐FTIR). Similar results have been observed in a previous human cohort study of 28 females, identifying aged skin as having significantly increased ‘freezable water’ in the SC, resulting in a decreased proportion of H_2_O molecules bound to NMF and ceramides. Consequently, as the skin ages, less moisture is bound within the SC structure, resulting in a lessened ability to maintain hydration levels [17]. The observation of skin dehydration, alongside significantly increased skin pH, concurs with other studies [18], and fits with the known increased prevalence of dry‐skin conditions in elderly individuals compared to younger adults [2].

Skin dehydration and pH increases are observed in those suffering from conditions such as ad [ 19], together with impaired skin barrier structure and function. As hydration and pH also increased with age, a significant association between TEWL and age could have been expected. However, a small decrease in TEWL from youngest to eldest was observed, indicative of minimal barrier function change. The results concur with similar work investigating the age‐related changes of skin barrier function, which saw no significant surface TEWL uplift with age [20]. A review of 167 in vivo studies reported a similar result and noted occasions of low‐level reduction in TEWL as age increased [21]. Elderly females appeared particularly susceptible to diminished skin health, with increased dryness, pH and TEWL_20_ compared to similarly aged males. Eczema incidence is greater in adult females vs. males [14], and the observed decline in skin health metrics for the eldest females could be linked to associations between menopause and skin barrier function shown previously [22].

Other observations suggest that structural changes within the SC in the elderly cause weakening of the barrier and increase susceptibility to skin damage, as well as diminished reparative properties. This has been observed in murine models where aged skin perturbed through skin tape‐stripping showed fewer tape strips required to cause damage and a 65% reduction in 24‐h recovery rate compared to younger skin [16]. In agreement with this study, TEWL_20_ was greatest in the oldest age group, indicative of significantly lower skin barrier integrity. Moreover, reactions to epicutaneously applied SLS, a common skin irritant, were associated with both age and skin barrier integrity, highlighting the relationship between advancing age and increased skin sensitivity (despite the tendency for reduced basal TEWL).

### 4.2 Lipidomic Profile Showed Significant Alterations Associated With Age and Xerosis

The application of ATR‐FTIR spectroscopy and lipidomic profiling alongside biophysical observations provides further insight into how structural alterations in the SC are associated with age‐related skin changes and increased susceptibility to dry‐skin conditions and perturbation.

Reduced skin hydration, measured through capacitance and increased visual dryness with age, were consistent with the spectroscopic characterisation of peaks associated with carboxylates (a proxy for NMF [23]) and SC amide I‐II ratio (SC hydration [24, 25]), with both reducing significantly from the 18–39 to 60+ age group. High pH, low stratum corneum hydration and reduced skin lipid levels have been previously identified as skin characteristics associated with one another [4], and are all shown to be impacted by age.

Lipid depletion was observed in the SC with advancing age, shown through significant peak area reductions in characterised total lipid (physiological and nonphysiological skin lipids [24, 26]) and lipid ester peaks (physiological esters and fatty acids associated with constituents of sebum such as wax esters and TAGs [27, 28]). The increased depletion of polyols and lipid esters in aged females vs. males again highlights the sex‐based physiological impacts of ageing and that loss of humectant properties through depletion of polyols like glycerine, exacerbated in females, is negatively impacting skin health. A review of literature in the area concluded that leave‐on humectant‐containing products are beneficial in maintaining skin health into old age [29].

This is consistent with other cohort studies' characterisation of SC lipids with increasing age [30]. Rogers et al.'s all‐female cohort study identified all major lipid types (fatty acids, ceramides and cholesterol) to broadly reduce with age in the skin, but particularly highlighted the depletion of ceramides as the most significant; except for CER[EOP], a more detailed analysis of ceramide composition was not undertaken. Reduced keratinocyte proliferation with age was thought to restrict the production of lipids [31]. Epidermal acid sphingomyelinase (A‐SMase) produces ceramides in the SC, which contribute to efficient healthy barrier function [6], and the inner epidermis sees A‐SMase deplete with age. Ceramidase activity has been observed as age‐independent, resulting in decreased ceramides in the inner epidermis as more ceramide is broken down than produced, consistent with skin losing reparative capability with age [32]. However, the outer epidermis maintains A‐SMase levels [32], resulting in the SC being better able to maintain ceramide levels than the inner epidermal layers.

Depletion of ceramides has also been observed in the lesional skin of AD patients with impaired skin barrier compared to healthy controls [33, 34]. Studies on nonlesional or uninvolved skin of AD patients have shown both ceramide depletion [35, 36] and no overall ceramide depletion [37, 38], despite the nonlesional AD skin barrier being impaired [39]. This suggests that an overall depletion of ceramides is not always required for barrier impairment, and points to changes in the relative abundance of the ceramide species and sub‐species which affect lipid lamellae formation [40]. Accordingly, we focused on the relative levels of ceramide sub‐species.

Lipidomic profiling showed TAGs to be the most abundant lipid class detected through mass spectrometry, and depletion of TAG with age was associated with lipid ester depletion. Previous works have shown TAG to be critical in functional skin barrier formation. Mutations relating to the reduction in catabolism or production of TAG have been linked to impaired barrier function and the development of conditions such as ichthyosis, characterised by skin xerosis. Another symptom accompanying xerosis is pruritus, and in studies of age‐associated pruritus, TAG depletion was again linked with reduced skin barrier function and disease onset [41]. Ma et al. also noted in their pruritus study that the NdS class of ceramides showed the greatest single association to barrier function [42]. The results of this study concur with these previous findings, with depletion and augmentation of TAG and CER[NdS] species, respectively, associated with an increase in age and advancing xerosis. However, the relative abundance of NH, NP, AH and AP observed here appears lower than in related literature [10, 43, 44, 45], but this could be due to this cohort exhibiting dry, eczema‐prone skin compared to the healthy participants previously studied in the cited works.

Recent developments in dermocosmetic formulations have included the addition of a greater variety of ingredients, such as ceramides, to better mimic the lipid profile of healthy skin and provide enhanced medicinal properties. Previous studies within this research group have shown ceramide‐containing formulations to possess greater skin barrier restorative properties compared to more simplistic products [46, 47]. Knowledge of ceramides and their impact on the maintenance of a functional skin barrier will help inform the development of optimal topical interventions. Here, stratification of the CER[NdS] profile showed changes in the proportions of sub‐species tending towards a reduction in acyl carbon chain length, whilst the abundance of sub‐species with longer sphingosine carbon chains increased with age. Studies have shown that those suffering from AD exhibit a greater presence of short‐chain ceramides (total chain length) compared to controls [48]. Ceramides exhibiting acyl chain lengths above 25 carbons have been directly linked to more ordered lipid organisation and are essential for healthy skin barrier function [6, 49, 50]. In a study of AD patients, the ratio between long (24ºC–32ºC) and short (14ºC–22ºC) acyl carbon chain lengths was significantly negatively associated with Staphylococcus spp. abundance, which is linked to skin dryness and AD severity, particularly in infants and the elderly [51, 52, 53]. Studies of individual subclasses of ceramides have reported similar findings, with the skin of infants who later develop AD showing upregulation of short 16ºC acyl chains compared to controls [54, 55], which concurs with the assessment of CER[NdS] observed here.

The profile of N‐ceramides as a whole significantly changed with age, with the proportion of CER[NH] decreasing whilst CER[NdS] increased. The association of NdS:NH ratio with age, dryness, TEWL_20_ and SC carboxylates (NMF [23]), coupled with a previously observed association of NdS:AS with AD and psoriasis, suggests N‐ceramides are a key biomarker associated with skin barrier homeostasis [56]. The SB in NH, NP, NS and NdS differ in their saturation and in the number of hydroxyl groups; both of these features impact the organisational packing order and flux of the lipid lamellae formed [57, 58]. Molecularly, increased prevalence of CER[NdS] instead of CER[NH] would result in more saturated lipids and fewer hydroxyl groups bound within the lipid structure. Given the association between TEWL_20_ and age, the changing N‐ceramide proportions appear to impact SC flux, which was significantly greater in the elderly after perturbation.

### 4.3 Limitations and Generalisability

Although our lipidomic analysis was comprehensive in breadth, quantifying 1385 lipid species, there are some lipid groups omitted, including free fatty acids and waxes. The study design did not include a healthy control group of participants with no history of eczematous skin conditions, which reduces the generalisability of results across the general population but focuses on biophysical alterations in individuals at greater risk who require intervention. Due to the inconsistent ratio of males to females in the study age groups, we have included statistical testing of both age and sex to determine their contribution to the observations. However, this resulted in smaller group sizes and reduced the strength of statistical comparisons, so the impact of sex on skin has not been discussed at length.

## 5 Conclusions

Within an at‐risk population who have previously suffered from AD, SC lipid levels undergo profound changes as the skin ages, with a broad depletion in total lipids, including triglycerides and an alteration in the distribution of ceramides. These changes, especially an increase in NdS ceramides with very short acyl chains, were associated with abnormal lamellar matrix formation, reduced skin barrier function and xerosis. These changes may contribute to the development of late‐onset AD, the re‐emergence of previously resolved AD and/or the changing presentation of the condition [59, 60].

The identification of significant biomolecules and SC structures that change with age is key for the future development of treatments for age‐associated skin conditions, like AD and asteatotic eczema. With formulations that help maintain optimum SC lamellar matrix structure and moisture levels, skin will not only appear younger for longer, but the onset and overall severity of dry‐skin conditions such as AD could be alleviated, helping to ease the burden of ageing populations on health services.

## Author Contributions

S.F.W.: methodology, formal analysis, visualisation, writing – original draft, writing – review and editing. P.A.: methodology, project administration, investigation, data curation, formal analysis, writing – review and editing. K.B.: project administration, investigation, writing – review and editing. J.C.: investigation, writing – review and editing. A.P.: project administration, investigation, writing – review and editing. A.Poy: project administration, investigation, writing – review and editing. M.J.C.: conceptualisation, supervision, writing – review and editing. S.G.D.: conceptualisation, methodology, writing – review and editing, supervision.

## Funding

This work was supported by L'Oreal Dermatological Beauty, UK, and CeraVe.

## Conflicts of Interest

Simon G Danby has received fees for giving lectures and/or attending advisory boards and research funding from Almirall, Astellas Pharma, Bayer Dermatology, Hyphens, Leo Pharma, L'Oreal, MSD, Pfizer, Rohto Pharma, Sanofi and Stiefel‐GSK. Michael J Cork has been/is a Clinical Trial Investigator for the following organisations: Atopix, Galapagos, Hyphens, Johnson & Johnson, Kymab, Leo, L'Oreal/La Roche Posay, Novartis, Pfizer, Regeneron and Sanofi‐Genzyme. He is an Advisory Board member, Consultant and/or invited lecturer for the following organisations: AbbVie, Amlar, Astellas, Atopix, Boots, Dermavant, Galapagos, Galderma, Hyphens, Johnson & Johnson, Kymab, Leo, L'Oreal/La Roche‐Posay, Menlo, Novartis, Oxagen, Pfizer, Procter & Gamble, Reckitt Benckiser, Regeneron, Sanofi‐Genzyme. Paul V Andrew, Samuel F. Williams, Κirsty Βrown, John Chittock, Abigail Pinnock and Anna Poyner declare no conflicts of interest.

## Supporting information


Supporting Information: S1.

## Data Availability

The data that support the findings of this study are available from the corresponding author upon reasonable request.
