# Supplementary material for: The Impact of Age on the Lipidomic Profile of the Stratum Corneum and Associated Effects on Structure, Function and Overall Skin Health in Adults Predisposed to Atopic Dermatitis
Source: Exp Dermatol. 2025 Dec 17;34(12):e70192. doi: 10.1111/exd.70192 (PMC12723634; doi:10.1111/exd.70192)
Supplement: Supplementary file 1 — Supporting Information: S1. [file EXD-34-e70192-s001.docx]

Supplementary Material: “The Impact of Age on the Lipidomic Profile of the stratum corneum and associated effects on Structure, Function and Overall Skin Health in adults predisposed to Atopic Dermatitis”

**Williams SF^1^, Andrew P^1^, Brown K^1^, Chittock J^1^, Pinnock A^1^, Poyner A^1^, Cork MJ^1,2^ and Danby SG^1^**

*^1^Sheffield Dermatology Research, Division of Clinical Medicine, School of Medicine & Population Health, University of Sheffield, Sheffield, UK; ^2^The Paediatric Dermatology Clinic, Sheffield Children’s Hospital, Sheffield, UK*

## Supplementary Methods

### Study Design & Setting

The study targeted recruitment of 58 participants, with a completion target of 48, stratified into 3 age groups of approximately equal size (18-39, 40-59, 60+). The completion target was calculated as participants required to detect a difference of 6 g/m 2 /h in TEWL between test sites after sodium lauryl sulfate (SLS) induced irritation with 82% power as this was the primary outcome of the main study.

### Eligibility criteria

Full inclusion and exclusion criteria are listed below:

Inclusion criteria:

1. Male or female, aged 18 years and over, falling into 1 of 3 age categories in line with recruitment targets: a. 18-39 b. 40-59 c. 60+
2. Volunteer understands the purpose, modalities and potential risk of the trial
3. Volunteers able to read and understand English
4. Volunteers willing to sign the informed consent
5. Volunteers with a self-reported history of eczema (lifetime). It was decided formal medical history to confirm previous AD diagnosis from a registered physician was not required. However, the following skin assessments were performed, with thresholds set to confirm presence of skin barrier deficiency and pass the study screening:
   1. Volunteers with dry skin, wherein visual dryness on the lower legs is ≥1 on a scale from 0-4.
   2. Volunteers with TEWL measuring ≥10 (≥7.5 for subjects aged 60+ years in accordance with reducing TEWL with advancing age^1^) on both distal volar forearms.

Exclusion criteria:

1. Volunteers currently undergoing, or requiring, active drug treatment for AD.
2. Volunteers with a known allergy/hypersensitivity to any of the excipients of the trial preparations (e.g. paraffin).
3. Volunteers who have used topical products (such as spray tan, emollient cream etc.) and/or abrasive cleansers/treatments (scrubs, bleach etc.) on the test sites for 1 week prior to and throughout the study (excluding the test products).
4. Volunteers with eczema, acne, suntan, hyperpigmentation, multiple nevi, tattoos, blemishes or dense body hair that obstruct the test areas.
5. Volunteers with perceptible differences between the visual scores (dryness or erythema) of the test sites at study enrolment as per Investigator’s assessment.
6. Volunteers with a condition that in the opinion of the investigator contradicts participation in the study.
7. Three or more bleach baths during any week within 4 weeks.
8. Volunteers who have used any medication that could interfere with the trial aim prior to and throughout the study
9. Volunteers currently participating in an interventional clinical trial.
10. Volunteer is incapable of giving fully informed consent.
11. Volunteers judged by the PI to be inappropriate for the trial.

### Skin Assessments

Measurements were collected from volar forearm and lateral lower leg sites (both right and left) before and after the 4-week treatment period. Four 5x4 cm skin sites were marked on each forearm, with the total 16x4 cm area centralised laterally and equidistant between the antecubital fossa and wrist. Two 5x4 cm skin sites were marked on each leg, with the 8x4 cm area placed to one side of the tibia and equidistant between the kneecap and ankle. Participants were asked not to apply any topical leave-on products and to abstain from waxing, shaving and epilating the arms or lower legs in the 7 days prior to study participation, and were permitted the use of the designated study products on skin test sites for the duration of the treatment period.

All biophysical measurements and visual skin assessments took place in the Skin Barrier Facility, with the study room temperature and humidity controlled to 20±2°C and 45±10% relative humidity. A period of 20-minutes acclimatisation was observed prior to skin assessments to allow skin sites to adjust to the local conditions.

### Visual Dryness

Skin dryness was graded by trained staff leading the clinical visit using the overall dry skin score (ODS^1^) scale from 0-4:

1. Absent.
2. Faint scaling, faint roughness and dull appearance.
3. Small scales in combination with a few larger scales, slight roughness, whitish appearance.
4. Small and larger scales uniformly distributed, definite roughness, possibly slight. redness and possibly a few superficial cracks.
5. Dominated by large scales, advanced roughness, redness present, eczematous changes and cracks.

### Skin Hydration

Four repeat measures of skin hydration were collected from lower leg test sites, using a Corneometer CM825 probe (CK Electronic GmbH, Cologne, Germany)

### Transepidermal Water Loss (TEWL)

Skin barrier function was indicated by quantification of transepidermal water loss (TEWL). Single measurements were collected from forearm sites with an Aquaflux TEWL machine (Biox Systems Ltd, UK).

### Skin Surface pH

Skin surface pH was recorded in triplicate from forearm sites with the Skin-pH-Meter 905 (CK electronic GmbH, Cologne, Germany).

### Skin Tape-Stripping (STS)

To enable study at an increased depth into the stratum corneum, skin tape-stripping was performed on the volar forearm using D-Squame cutaneous stripping discs (CuDerm Corporation, Dallas, USA). 225 g cm^-2^ of pressure was applied to each strip after application, before immediate removal. A total of 20 strips were applied and removed at a single forearm site before and after the treatment period.

(SquameScan 850A, CuDerm Corporation, Dallas, USA). Equating 100μm/cm2 protein to 1.9±2 μm of SC removed by STS allowed the measurement depth to be estimated, using methodology from previously published studies.^2,3^

### Skin Sensitivity Testing

Skin sensitivity testing Finn Chambers (12mm aluminium chambers on Scanpor tape) containing a filter insert and 50μL 1% w/v SLS were applied to designated test sites for 24 hours (SmartPractice, Phoenix, AZ, USA). Further skin assessments were conducted 24±2 hours after patch removal.

### Lipidomic Sampling & Analysis

Samples of skin material were collected from the forearm test sites at baseline and at the end of treatment for lipidomic analysis. Each sample was collected using a triplicate of 14 mm D-squame discs (D101, CuDerm Corporation, Dallas, USA). Five consecutive tape strips were collected at each site with the first sample discarded to remove surface contaminants. For each subsequent layer the discs were applied to the skin under pressure from the plunger (CuDerm Corporation) for 15 seconds. Samples were stored at -20°C. Samples for tape-strip layers 2 and 5 were sent to Lipotype GmbH (Dresden, Germany) for quantification of absolute skin lipid abundances using high resolution Orbitrap mass spectrometry.^4^ Lipids were extracted from tape-strips using chloroform and methanol. Class specific internal standards were added to the samples for calibration which were then dried and resuspended in 7.5 mM ammonium acetate in chloroform:methanol:propan-2-ol. Mass spectra were acquired on a hybrid quadrupole/Orbitrap mass spectrometer equipped with a nano-flow electrospray ion source in both positive and negative ion mode. Lipid abundances were normalised to protein based on opacity of the collected discs measured with D-squame scan 850A. Lipid abundance data is presented here normalised to the amount of protein on a D-squame disc from the same tape-stripping layer.

### ATR-FTIR Spectroscopy

Mid-infrared spectra were collected from volar forearm sites using the Agilent 4300 Fourier Transform Infrared (FTIR) Spectrometer, fitted with a 3-Bounce-2-Pass attenuated total reflectance (ATR) scanning interface. The spectrometer was equipped with a deuterated triglycine sulphate (DTGS) detector (Agilent Technologies, Santa Clara CA, US). A one-hour warm-up period was implemented to allow internal components and temperature to stabilise prior to measurement collection. Two replicate measurements were collected from the surface of the skin on right and left forearm sites, with single measurements collected at each tape-strip interval (STS: 5, 10, 15, 20). Each collected spectrum consisted of an average of 32 scans collected (32 background scans were also collected prior to each measurment) at 4 wavenumber resolution. Quantification of peak intensity and location done using Omnic 9.0 software (Thermo Electro Corp., Madison, USA). All quantitative peak intensities were relative to the Amide II bond of protein, at 1550 cm^-1^ to account for different sample-interface contact between measurements. Spectral processing was conducted using Spectrogryph (v1.2.15, 2020).

### Data Structuring

All absolute (pmol) lipid quantifications were normalised to the total amount of protein (µg) present on the skin tape-strip when extracting lipids from volar forearm sites, to account for variance in total amount of sample collected from the test site. Prior to linear model creation, an acceptance threshold of 75% was used to exclude species present in a minority of participants, and where abundance of lipids fell below the limit of detection, median values were imputed from within each age group to fill blank dataspaces.

# References

1. Serup, J. EEMCO guidance for the assessment of dry skin (xerosis) and ichthyosis: clinical scoring systems. *Skin Res Technol* **1**, 109–114 (1995).

2. Voegeli, R., Rawlings, A. V., Doppler, S., Heiland, J. & Schreier, T. Profiling of serine protease activities in human stratum corneum and detection of a stratum corneum tryptase-like enzyme. *Int J Cosmet Sci* **29**, 191–200 (2007).

3. Boncheva, M., Damien, F. & Normand, V. Molecular organization of the lipid matrix in intact Stratum corneum using ATR-FTIR spectroscopy. *Biochim Biophys Acta Biomembr* **1778**, 1344–1355 (2008).

4. Sadowski, T. *et al.* Large-scale human skin lipidomics by quantitative, high-throughput shotgun mass spectrometry. *Sci Rep* **7**, (2017).

#

## Supplementary Results

### Tables

Table S1: 15 most significant lipid-age associations based on individual t-test age group comparison, sorted by ascending p-value.

| **Subspecies** | **Age Comparison** | **Difference** | **SE of difference** | **Fold Change** | **P value** |
| --- | --- | --- | --- | --- | --- |
| **NdS 24:0;2/16:0;0** | 18-39 vs 60+ | 0.37 | 0.04 | 2.35 | 8.00E-14 |
| **TAG 45:0;0** | 18-39 vs 40-59 | -0.36 | 0.05 | 2.27 | 2.73E-10 |
| **TAG 46:1;0** | 18-39 vs 60+ | -0.44 | 0.06 | 2.76 | 4.28E-09 |
| **TAG 46:2;0** | 18-39 vs 60+ | -0.43 | 0.07 | 2.71 | 9.38E-09 |
| **NdS 22:0;2/23:0;0** | 18-39 vs 60+ | 0.19 | 0.03 | 1.56 | 1.10E-08 |
| **TAG 45:1;0** | 18-39 vs 40-59 | -0.37 | 0.06 | 2.36 | 2.02E-08 |
| **TAG 47:3;0** | 18-39 vs 40-59 | -0.37 | 0.06 | 2.34 | 4.56E-08 |
| **TAG 45:1;0** | 18-39 vs 60+ | -0.42 | 0.07 | 2.62 | 4.73E-08 |
| **TAG 48:2;0** | 18-39 vs 60+ | -0.43 | 0.07 | 2.70 | 5.55E-08 |
| **TAG 49:0;0** | 18-39 vs 40-59 | -0.31 | 0.05 | 2.06 | 5.58E-08 |
| **TAG 44:3;0** | 18-39 vs 40-59 | -0.29 | 0.05 | 1.95 | 6.06E-08 |
| **NdS 24:0;2/16:0;0** | 40-59 vs 60+ | 0.28 | 0.04 | 1.90 | 7.58E-08 |
| **TAG 47:1;0** | 18-39 vs 40-59 | -0.37 | 0.06 | 2.34 | 8.39E-08 |
| **TAG 46:1;0** | 18-39 vs 40-59 | -0.34 | 0.06 | 2.21 | 9.32E-08 |
| **NdS 20:0;2/25:0;0** | 18-39 vs 60+ | 0.23 | 0.04 | 1.69 | 9.47E-08 |

Table S2: 15 most significant lipid-age associations (after removal of colinear species) based on individual t-test age group comparison, sorted by ascending p-value. Collinearity determined by Pearson’s correlation, with lipids excluded if correlation with a lipid exhibiting a smaller p value exceeded r = 0.8.

| **Subspecies** | **Age Comparison** | **Difference** | **SE of difference** | **Fold Change** | **P value** |
| --- | --- | --- | --- | --- | --- |
| **NdS 24:0;2/16:0;0** | 18-39 vs 60+ | 0.37 | 0.04 | 2.35 | 8.00E-14 |
| **TAG 45:0;0** | 18-39 vs 40-59 | -0.36 | 0.05 | 2.27 | 2.73E-10 |
| **NdS 22:0;2/23:0;0** | 18-39 vs 60+ | 0.19 | 0.03 | 1.56 | 1.10E-08 |
| **NdS 24:0;2/16:0;0** | 40-59 vs 60+ | 0.28 | 0.04 | 1.90 | 7.58E-08 |
| **NdS 20:0;2/23:0;0** | 18-39 vs 60+ | 0.17 | 0.03 | 1.49 | 1.95E-07 |
| **DAG 16:1;0_18:2;0** | 40-59 vs 60+ | 0.29 | 0.05 | 1.97 | 4.42E-07 |
| **NdS 22:0;2/28:0;0** | 18-39 vs 60+ | 0.24 | 0.04 | 1.76 | 4.79E-07 |
| **TAG 45:0;0** | 18-39 vs 60+ | -0.28 | 0.06 | 1.89 | 6.11E-06 |
| **NdS 18:0;2/28:0;0** | 18-39 vs 60+ | 0.17 | 0.03 | 1.47 | 7.90E-06 |
| **NdS 24:0;2/22:0;0** | 18-39 vs 60+ | 0.13 | 0.03 | 1.34 | 1.41E-05 |
| **NdS 26:0;2/16:0;0** | 18-39 vs 60+ | 0.18 | 0.04 | 1.50 | 1.68E-05 |
| **TAG 41:0;0** | 18-39 vs 40-59 | -0.25 | 0.05 | 1.77 | 1.84E-05 |
| **AdS 19:0;2/27:0;1** | 18-39 vs 60+ | 0.19 | 0.04 | 1.55 | 3.04E-05 |
| **AP 18:0;3/26:0;1** | 18-39 vs 60+ | 0.18 | 0.04 | 1.50 | 3.30E-05 |
| **DAG 15:1;0_18:1;0** | 40-59 vs 60+ | 0.16 | 0.04 | 1.44 | 4.09E-05 |

### Figures


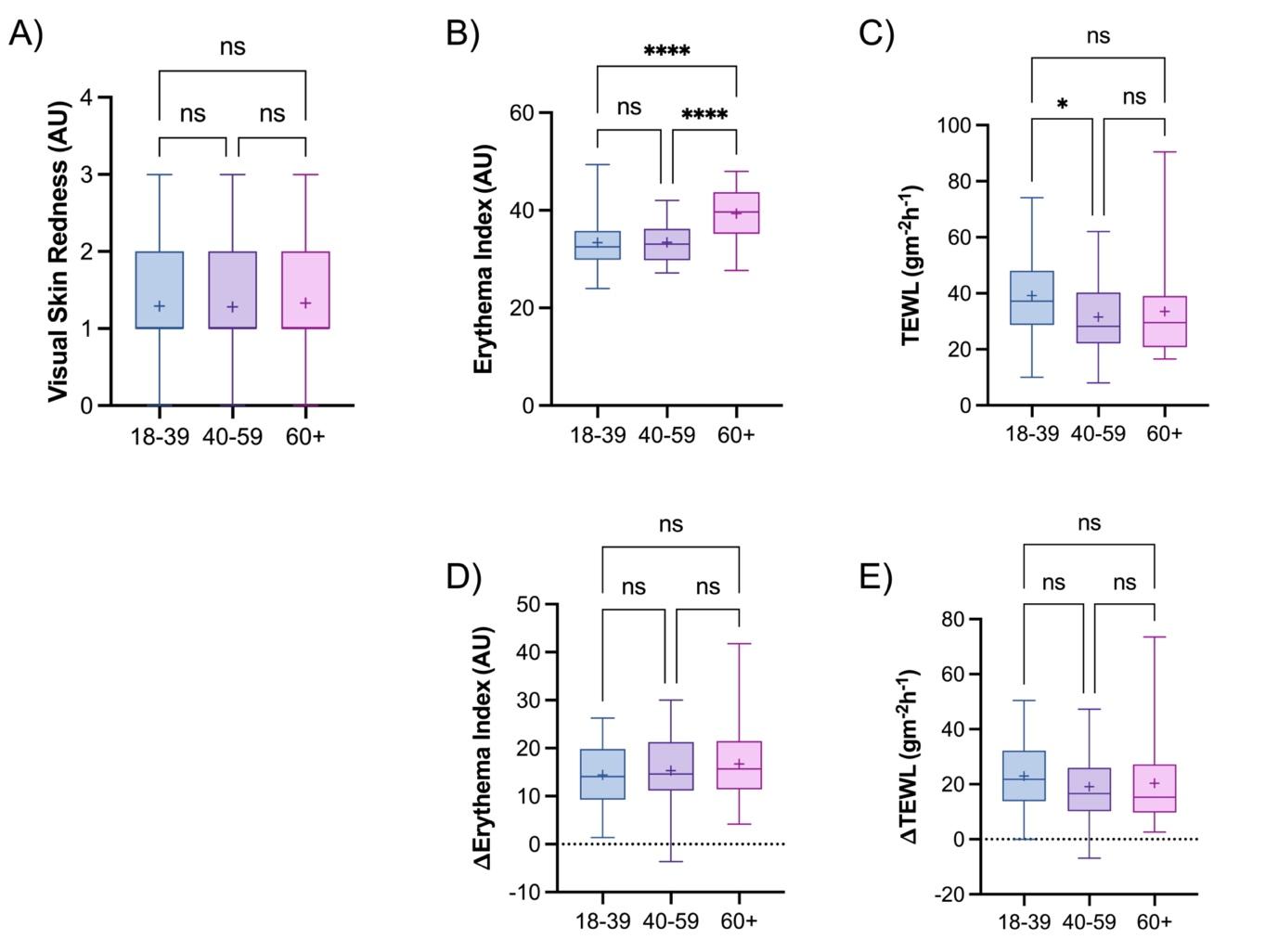


Figure S1: Skin sensitivity testing assessed through post-exposure; visual erythema (A), erythema index (B), transepidermal water loss (TEWL) (C) and pre-to-post-exposure change in; erythema (D) and TEWL (E) after exposure to sodium lauryl sulphate. Box-and-whisker plots depict the minimum, lower quartile, median, upper quartile and maximum values present in each group with “+” indicating the mean value. Asterisks indicate the results of one-way ANOVA with post-hoc Tukey HSD statistical testing (ns = not significant, *p<0.05, **p<0.01, ***p<0.001, ****p<0.0001).


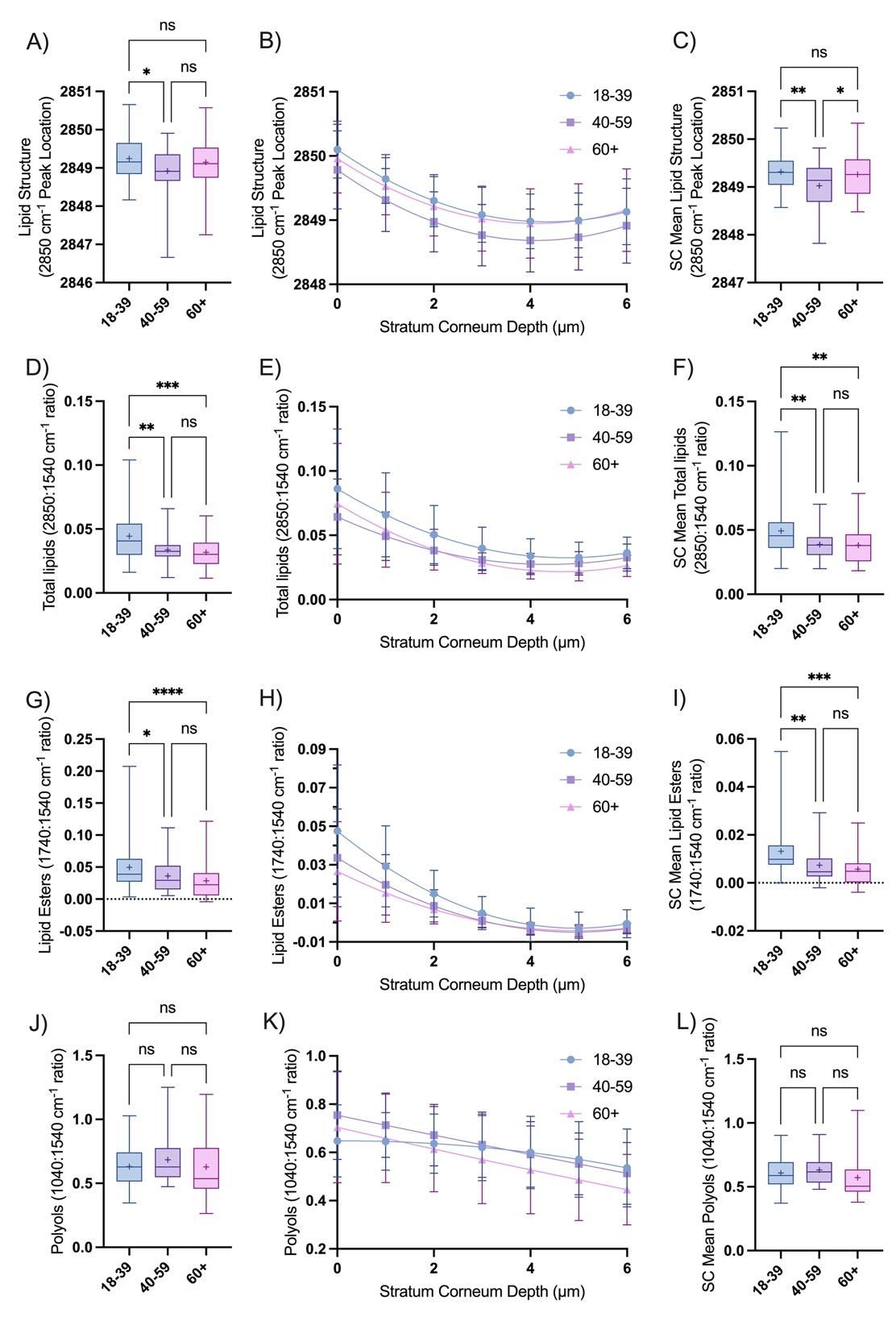


Figure S2: Age associated lipid composition changes in the stratum corneum. Measurement of lipid structure (A-C), total lipids (D-F), lipid esters (G-I) and polyols (J-L) through peak area relative to Amide II by ATR-FTIR. Spectroscopic parameters were measured after five serial tape-strips (A,D,G,J), after every 5 tape-strips from 0-20 tape-strips adjusted to stratum corneum depth (B,E,H,K) and as an average across 6μm of SC (C,F,I,L). Box-and-whisker plots depict the minimum, lower quartile, median, upper quartile, and maximum values present in each group. “+” indicates the mean value. Outliers plotted as individual points. Asterisks indicate the results of a one way ANOVA with post-hoc Tukey HSD (ns = not significant, *p<0.05, **p<0.01,***p<0.001, ****p<0.0001).


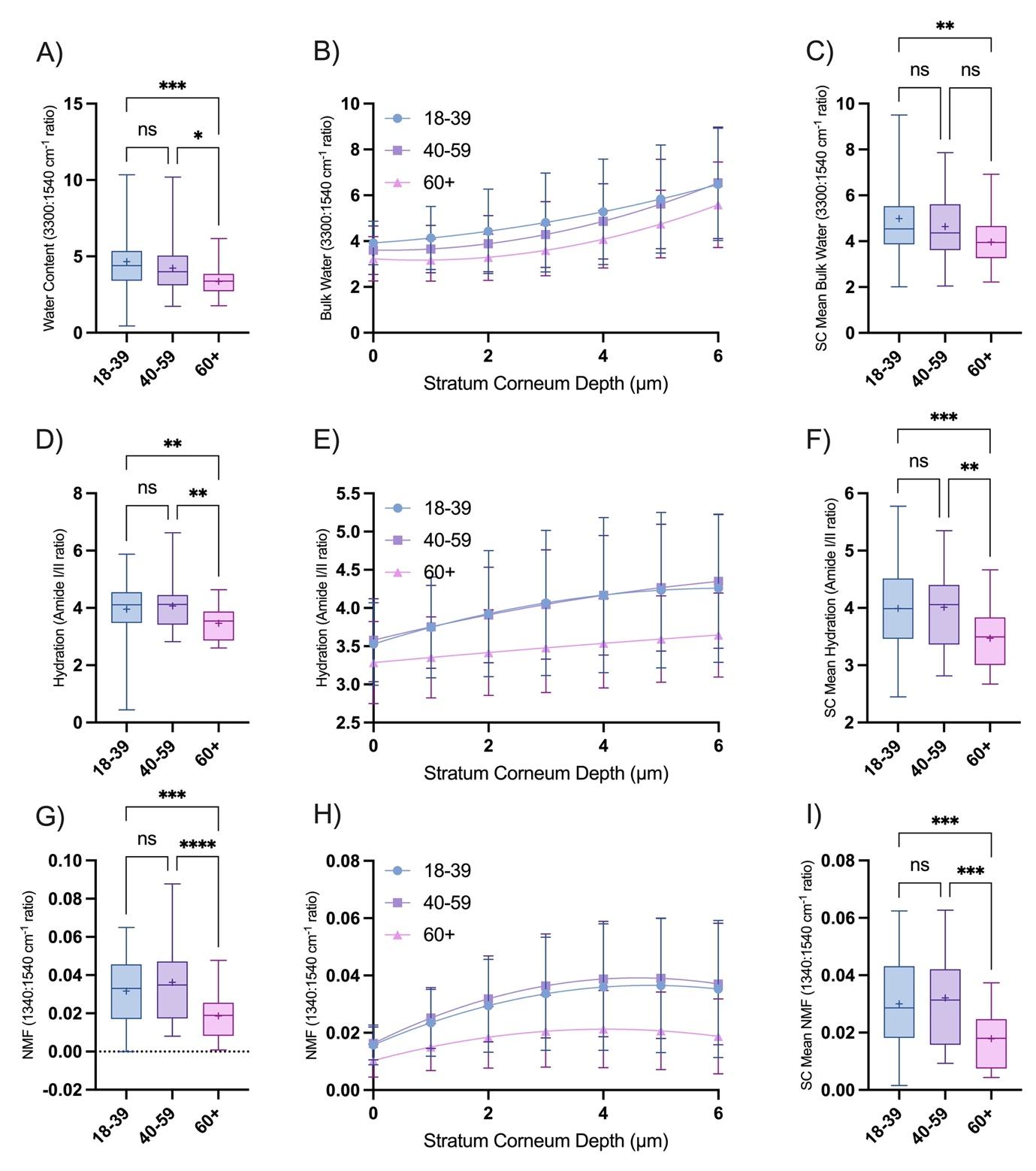


Figure S3: Age associated non-lipid composition changes in the stratum corneum. Measurement of bulk-water content (A-C), SC hydration (D-F), carboxyl groups (G-I) through peak area relative to Amide II by ATR-FTIR. Spectroscopic parameters were measured after five serial tape-strips (A,D,G), at 5 tape-strip from 0-20 tape strips adjusted to stratum corneum depth (B,E,H) and as an average across 6μm of SC (C,F,I). Box-and-whisker plots depict the minimum, lower quartile, median, upper quartile, and maximum values present in each group. “+” indicates the mean value. Outliers plotted as individual points. Asterisks indicate the results of a one way ANOVA with post-hoc Tukey HSD (ns = not significant, *p<0.05, **p<0.01,***p<0.001, ****p<0.0001).


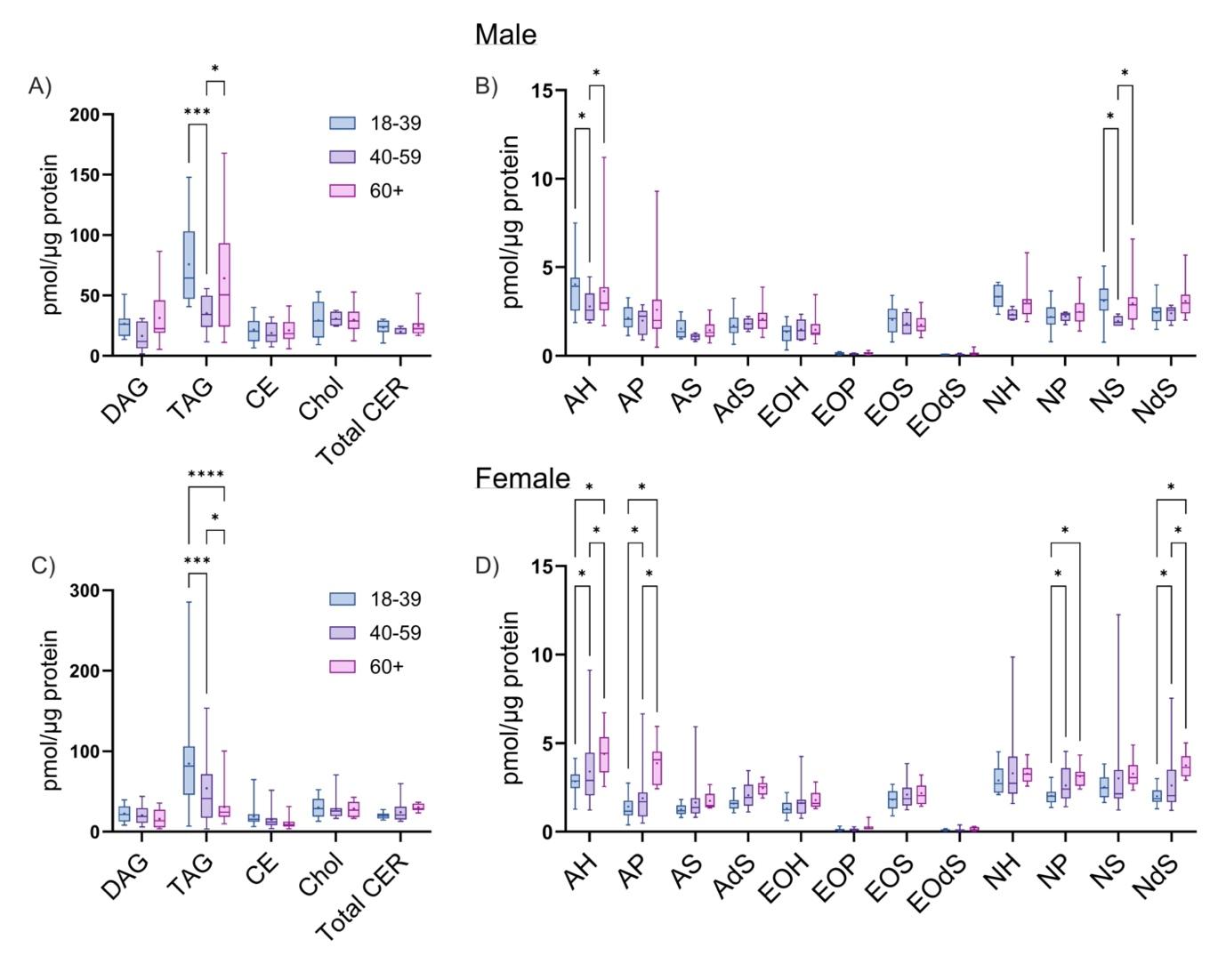


Figure S4: Top-level lipidomic analysis extracted from second skin tape-strip (STS) removed from volar forearm sites of Male (A & B) or Female (C & D) participants; Age associated changes in stratum corneum lipid groups (A & C) and ceramide (B & D) levels, relative to protein. Asterisks indicate the results of a one way ANOVA with post-hoc Tukey HSD (ns = not significant, *p<0.05, **p<0.01,***p<0.001, ****p<0.0001).


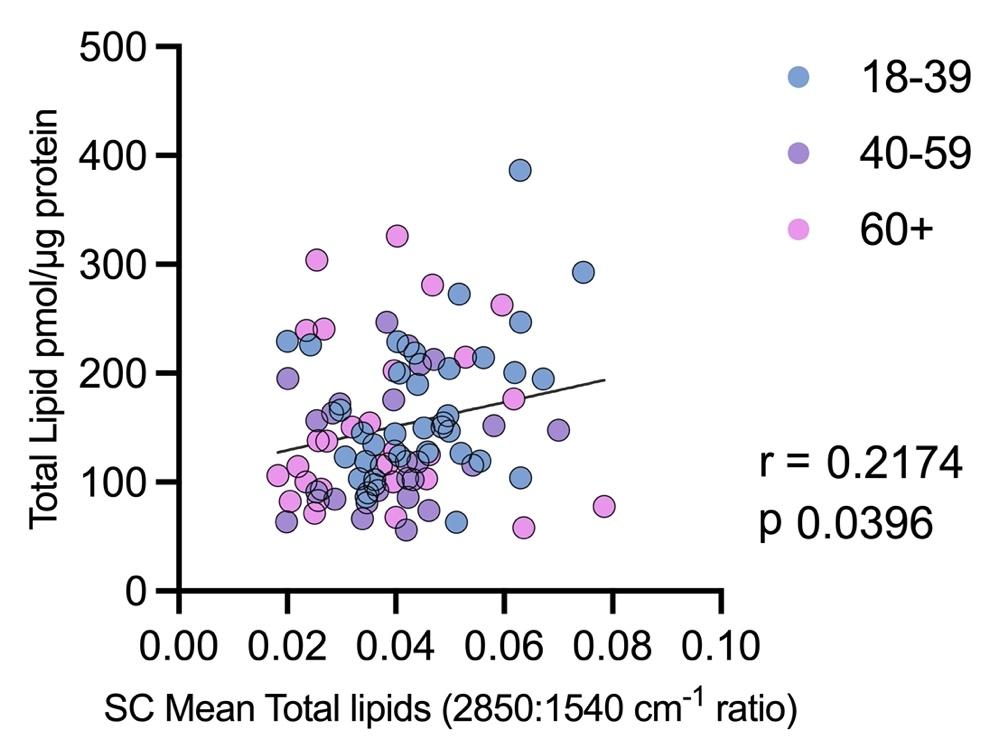


Figure S5: Pearson correlation between total lipid extracted from skin tape-strip 2 (STS 2) quantified via Orbitrap mass spectrometry and ATR-FTIR derived total lipid present on volar forearm skin sites. Colours indicate participant cohort stratification by age group.


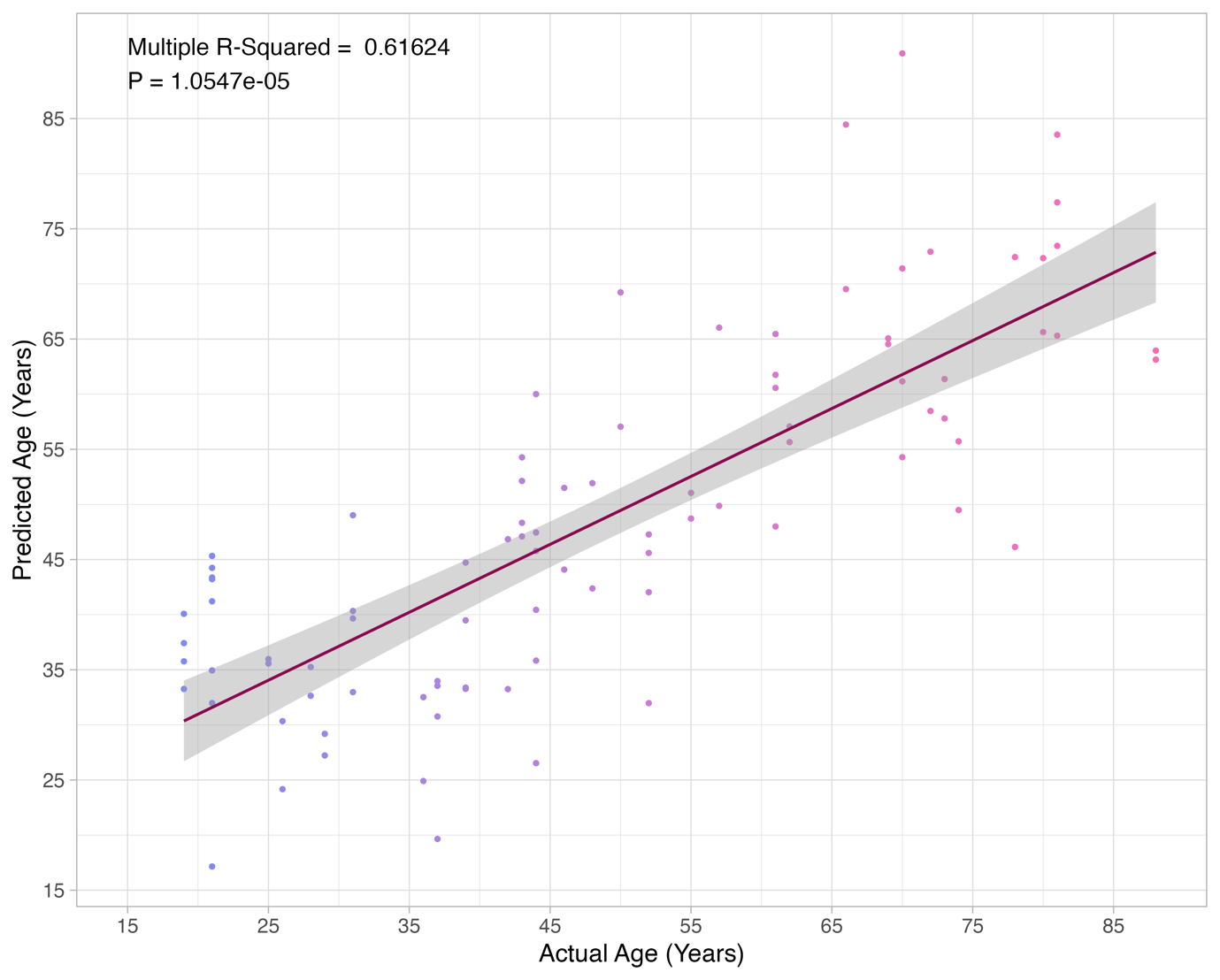


Figure S6: Predicted vs Actual age of the study cohort. Age predictions were computed through a linear regression model based on absolute quantities of 382 lipids defined by mass spectrometry. Plot annotated with Pearson’s correlation line of best fit and 95% confidence interval shaded area.
